# Elevated and cross‐responsive CD1a‐reactive T cells in bee and wasp venom allergic individuals

**DOI:** 10.1002/eji.201545869

**Published:** 2015-11-17

**Authors:** Sumithra Subramaniam, Aamir Aslam, Siraj A. Misbah, Mariolina Salio, Vincenzo Cerundolo, D Branch Moody, Graham Ogg

**Affiliations:** ^1^ MRC Human Immunology UnitWeatherall Institute of Molecular Medicine and University of Oxford, NIHR Biomedical Research CentreOxfordEnglandUK; ^2^ Section of Musculoskeletal DiseaseUniversity of LeedsLeedsUK; ^3^ Department of Clinical ImmunologyOxford University Hospitals NHS TrustOxfordUK; ^4^ Division of RheumatologyImmunology and AllergyDepartment of MedicineBrigham and Women's HospitalHarvard Medical SchoolBostonMAUSA

**Keywords:** CD1a‐reactive T cells, Immunotherapy, Phospholipase, Venom allergy

## Abstract

The role of CD1a‐reactive T cells in human allergic disease is unknown. We have previously shown that circulating CD1a‐reactive T cells recognize neolipid antigens generated by bee and wasp venom phospholipase, and here tested the hypothesis that venom‐responsive CD1a‐reactive T cells associate with venom allergy. Circulating T cells from bee and wasp venom allergic individuals, before and during immunotherapy, were exposed to CD1a‐transfected K562 cells in the presence of wasp or bee venom. T‐cell response was evaluated based on IFNγ, GM‐CSF, and IL‐13 cytokine production. Venom allergic individuals showed significantly higher frequencies of IFN‐γ, GM‐CSF, and IL‐13 producing CD1a‐reactive T cells responsive to venom and venom‐derived phospholipase than healthy individuals. Venom‐responsive CD1a‐reactive T cells were cross‐responsive between wasp and bee suggesting shared pathways of allergenicity. Frequencies of CD1a‐reactive T cells were initially induced during subcutaneous immunotherapy, peaking by weeks 5, but then reduced despite escalation of antigen dose. Our current understanding of venom allergy and immunotherapy is largely based on peptide and protein‐specific T cell and antibody responses. Here, we show that lipid antigens and CD1a‐reactive T cells associate with the allergic response. These data have implications for mechanisms of allergy and approaches to immunotherapy.

## Introduction

Vespula (wasp) and apis (bee) stings cause different clinical reactions in individuals, ranging from the expected localized cutaneous inflammation that is directly mediated by venom and seen in virtually all humans to systemic host response anaphylaxis and death in venom‐allergic individuals 1. The secondary allergic immune responses are mediated by allergen‐specific IgE antibodies, mast cells, eosinophils, and associated with peptide‐specific type 2 T‐cell responses. Venom allergic individuals have elevated frequencies of venom peptide‐specific T cells circulating in peripheral blood 2, 3, 4, 5. In other cases peptide‐specificity is unknown or assumed. During venom‐specific immunotherapy, repeated exposure to the venom in a controlled environment leads to allergen desensitization and clinical tolerance induction. Successful immunotherapy has been associated with a rise in allergen‐specific blocking IgG antibodies (IgG4) 6 and a decrease in Th2 cytokines from peptide‐specific CD4+ T cells 7. This transition from a Th2 cytokine profile bias affects IgE class switching and eosinophil differentiation and survival, which are Th2 cytokine‐dependent 8. Regulatory T cells have also been implicated in successful desensitization, with an increase in peptide antigen‐specific CD4+CD25+ cells and IL‐10, potentially facilitating IgG4 production 2, 9. However, the underlying mechanisms by which allergen immunotherapy achieves clinical improvement in allergic patients are still not fully understood.

The CD1 family of antigen presenting molecules (CD1a, CD1b, CD1c, CD1d) are cell surface glycoproteins expressed on cells capable of presenting lipid antigen 10. They have significant structural similarities to MHC class I molecules, including a heavy chain that folds to form a hollow antigen‐binding cleft and noncovalent association with β2‐microglobulin. However, CD1 proteins are encoded outside the MHC and are minimally polymorphic 11. CD1 proteins bind and present lipid antigens, and there are now a growing number of known antigens recognized by T cells, including sphingolipids and phospholipids 12. For these amphipathic lipids, the alkyl chains lie within the cleft of CD1, while polar head groups extend out and interact with the TCR 13, 14. More recently, smaller headless skin oils and related self‐lipid antigens were also shown to bind CD1a and stimulate T cells, where the TCR binds to CD1a without interacting directly with the lipid ligand 15, 16.

CD1a is expressed on thymocytes and several APCs in the periphery, but most CD1a expression is found in the skin, where epidermal Langerhans cells (LCs) express CD1a at particularly high density. CD1a is also expressed by subsets of dermal DCs and DCs at other mucosal sites, including the lungs 17, 18. Indeed, high intensity CD1a expression has long been used as a marker of LCs 18. Polyclonal CD1a‐autoreactive T cells are a distinct and normal component of the T‐cell repertoire and are found in most humans 19, 20. They were identified in both peripheral blood and skin, expressing skin homing markers, such as cutaneous lymphocyte antigen 19. Moreover, comparison of CD1a autoreactive T cells to several cell types and organs found that antigenic oils specifically accumulate in the skin 16. The colocalization of CD1a autoreactive T cells, CD1a proteins, and CD1a‐presented antigens within the skin suggests possible roles in inflammatory skin disease or homeostatic immunity of the skin.

While the recognition of lipid antigens by T cells is well established in healthy donors 16, 19, 20, 21, the role of CD1‐reactive lipid antigens and T cells in disease is largely unstudied. Nevertheless, CD1‐reactive T cells have been implicated in the host defense against pathogens, autoimmune, and anti‐tumor responses 22, 23. Studies have shown that CD1d‐restricted T cells may also contribute to the immune response in allergy. For example, mice deficient in NKT restricted to CD1d, did not develop allergen‐induced airway hyper‐reactivity 24. Lipids, particularly phospholipids, at the pollen surface were shown to bind CD1d and CD1a and were recognized by T cells with highest responses seen in sensitive subjects at the peak of the allergic season 25. However, most studies have focused on CD1d‐restricted lipids, as only CD1d is expressed in mice, but new tools, including MHC^low^ CD1‐high human APCs now allow cross‐sectional clinical studies in humans 19, 21

Because wasp and bee venom are normally injected near the site of CD1a expression, we recently investigated wasp and bee venom for the presence of CD1a‐presented antigens using T‐cell clones and polyclonal peripheral blood derived T cells from healthy individuals 21. Venom contains a number of different active proteins and peptides designed to paralyze prey or parasite hosts or deter predators by causing pain. Some of the proteins, including phospholipase and hyaluronidase, are well known to induce protein‐specific B and T‐cell responses leading to clinical allergic reactivity in susceptible individuals. However, we found CD1a autoreactive cells responding to neolipid antigens. That is, wasp venom phospholipase A1 (PLA1) and bee venom phospholipase A2 (PLA2) act within the skin on intact nonantigenic phospholipids to generate antigenic fatty acids and lysophospholipids 21. This study suggested that T‐cell responses reactive to CD1a may contribute to the ensuing inflammatory response that is seen nearly universally in humans. However, CD1a‐reactive cells have not been investigated as a possible cause of the exaggerated response seen in venom allergic disease.

To study CD1a‐reactive polyclonal human T cells, we used APCs lacking detectable surface expression of MHC proteins (K562 cells), which minimizes MHC alloreactive responses. K562 cells were transfected to express CD1a (K562‐CD1a) at high density. This can replicate the use of primary CD1a‐expressing antigen presenting cells, allowing us to compare polyclonal CD1a‐reactive T‐cell responses to venom in unrelated healthy and venom allergic individuals 21 We have demonstrated an increase in venom‐responsive CD1a‐reactive T cells with a Th2 bias in allergic compared to nonallergic individuals, and eventual loss of the T cells during acquisition of clinical tolerance, providing evidence for contribution of CD1a and lipids to the allergic response in humans.

## Results

### Increased bee venom‐responsive CD1a‐reactive T cells in venom allergic individuals

**Figure 1:**
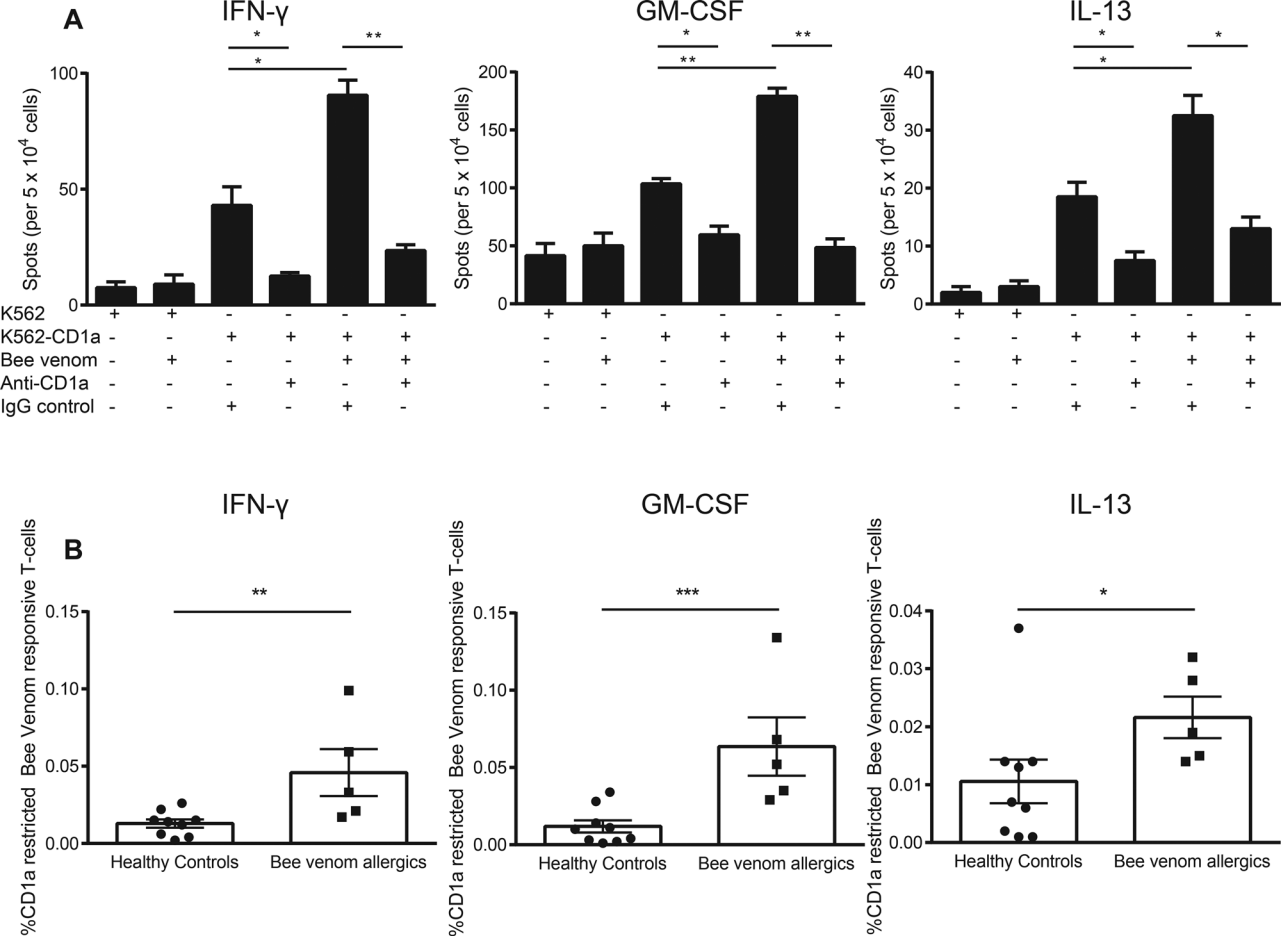
Bee allergic individuals show increased bee venom responsive CD1a‐reactive T cells compared to nonallergic individuals. CD3+ T cells were isolated from peripheral blood of nonallergic (*n* = 8) and bee allergic individuals (*n* = 5) by magnetic bead separation. (A) CD1a reactivity was examined by ELISpot with K562 or K562‐CD1a in the presence or absence of bee venom (1 μg/mL) and/or 10 μg/mL anti‐CD1a mAb (OKT6). Data bars are shown as mean ± SEM and are from 1 allergic donor out of five studied. (B) Frequency of CD1a‐reactive T cells responsive to bee venom above the auto‐reactive response. Data are shown as mean ± SEM and are pooled from 13 independent experiments, each performed in duplicate. *p < 0.05; **p < 0.01; ***p < 0.001; unpaired nonparametric *t* test.

We recruited adult individuals with a history of anaphylaxis to bee venom, and a positive skin prick test or raised bee venom‐specific IgE antibodies, as well as healthy controls. To understand the role of lipid antigens in venom allergy, first we investigated cytokine production by venom‐responsive, CD1a‐reactive T cells isolated ex vivo from our allergic and control cohorts. We used K562 cells either mock‐transfected or CD1a‐transfected as a universal target cell system, as they express low levels of MHC‐class I and II, and have been shown to accurately reflect responses observed with monocyte‐derived CD1a‐positive cells, CD34‐derived LC‐like cells, and skin‐derived CD1a‐positive cells 19, 21. We detected low ex‐vivo IFN‐γ, GM‐CSF, and IL‐13 responses to mock transfected K562 cells, in the presence or absence of bee venom. In the absence of venom, we detected background response to K562 cells at a rate of ∼6 per 10 000, which is similar to that previously reported and likely reflects low level MHC alloreactivity or NK‐cell response 19, 21. T‐cell autoreactive responses to K562‐CD1a in the absence of bee venom were higher than to mock transfected K562, at ∼ 1:350 T cells, in agreement with previous studies showing circulation of CD1a autoreactive T cells in vivo. This response is thought to be due to recognition of K562‐derived lipids 19, 20. However, in the presence of bee venom, significantly increased responses were observed only in the presence of CD1a‐transfected cells, consistent with the conclusion that CD1a mediates the response. Strong evidence for an essential role of CD1a was found when the IFN−γ response were reduced nearly to background levels by a blocking anti‐CD1a antibody (OKT6) but not isotype control (Fig. 1A left panel). Similar patterns of response were seen with GM‐CSF (Fig. 1A, middle panel) and IL‐13 (Fig. 1A, right panel) in response to bee venom and CD1a expressing targets. The frequency of IFN‐γ (*p* < 0.01; Fig. 1B, left panel), GM‐CSF (*p* < 0.001; Fig. 1B, middle panel), and IL‐13 (*p* < 0.05; Fig. 1B, right panel) responding T cells in the presence of K562‐CD1a and bee venom was greater in a panel of bee venom allergic than nonallergic individuals (Fig. 1B). These responses show that T‐cell responses to bee venom are in part mediated by CD1a, and are increased in bee venom allergic compared to nonallergic individuals.

### Bee venom PLA2 reproduces the CD1a‐reactive whole venom response in allergic individuals

**Figure 2:**
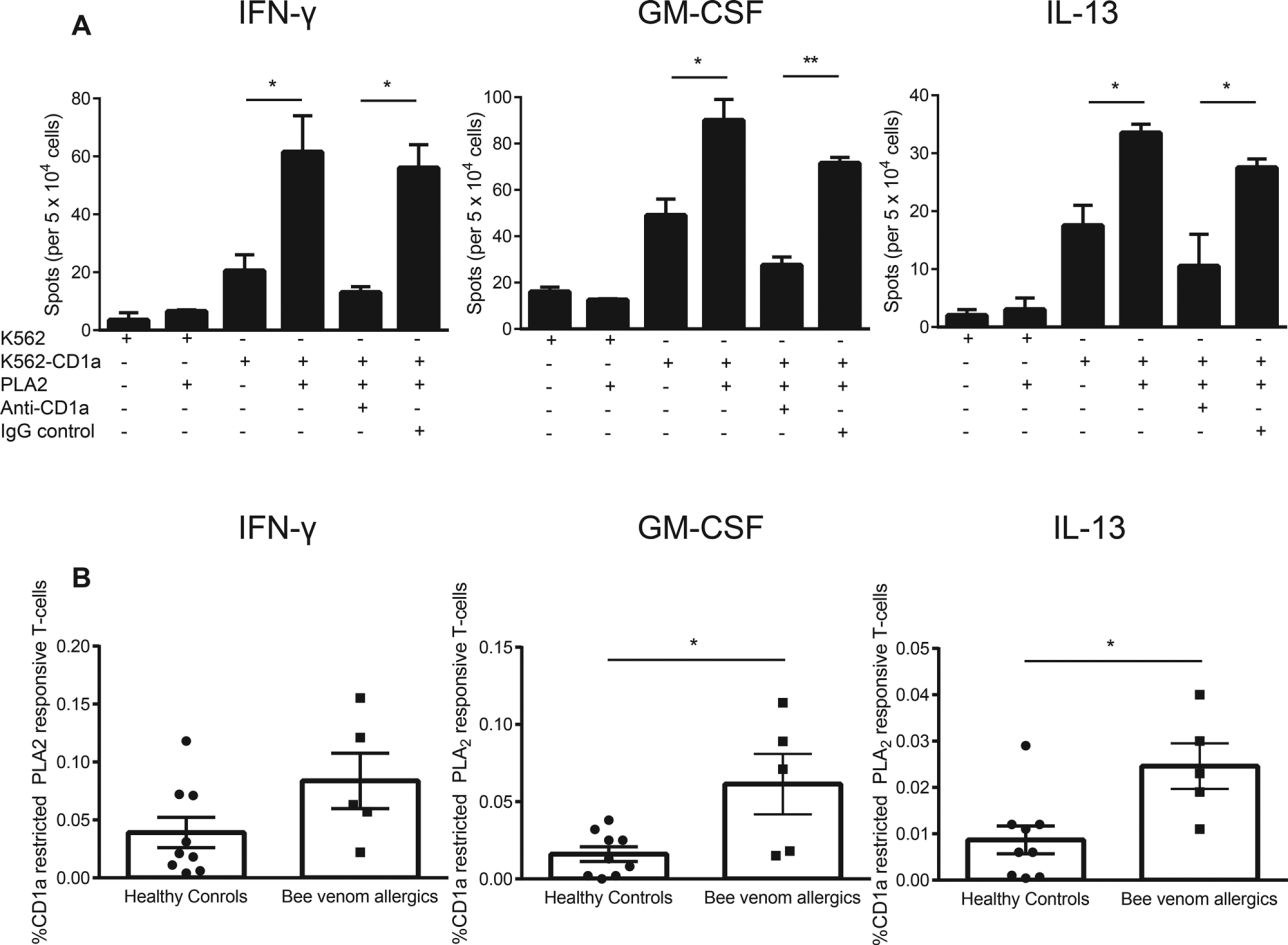
Bee allergic individuals show increased frequencies of CD1a‐reactive T cells responsive to bee venom PLA2 compared to nonallergic individuals. CD3+ T cells were isolated from peripheral blood of nonallergic (*n* = 9) and bee allergic individuals (*n* = 5) by magnetic bead separation. (A) CD1a reactivity was examined by ELISpot with K562 or K562‐CD1a in the presence or absence of bee venom PLA2 (1 μg/mL) and/or 10 μg/mL anti‐CD1a mAb (OKT6). Data bars are shown as mean ± SEM and are from one allergic donor of five studied. (B) Frequency of CD1a‐reactive T cells responsive to bee venom PLA2 above the autoreactive response. Data are shown as mean ± SEM and are pooled from 14 independent experiments, each performed in duplicate. *p < 0.05; **p < 0.01; unpaired nonparametric *t* test.

Phospholipase (PLA) is known to be an important target for peptide‐specific T cells in venom allergic individuals 2, 3, 4, 5. Previously, we have shown that PLA2 in bee venom can generate CD1a lipid antigens for recognition by CD1a‐reactive T cells in cultured assays of T cells derived from healthy donors 21. We therefore sought to determine if the increased T‐cell responses to bee venom in allergic individuals were also generated by PLA2 itself or whether other pathways were important in allergy. In the presence of PLA2 and K562‐CD1a, ex‐vivo T cells produced IFN‐γ, GM‐CSF, and IL‐13 (Fig. 2A). Responses were CD1a‐reactive as the T‐cell responses to PLA2 were abrogated in the presence of a blocking anti‐CD1a antibody but not an isotype control (Fig. 2A). The frequency of IFN‐γ (ns; Fig. 2B, left panel), GM‐CSF (*p* < 0.05; Fig. 2B, middle panel), and IL‐13 (*p* < 0.05; Fig. 2B, right panel) producing T cells in the presence of K562‐CD1a and PLA2 above the autoreactive response, was greater in bee venom allergic than nonallergic individuals. Thus, the increase in IFN‐γ, GM‐CSF, and IL‐13 producing CD1a‐reactive T cells in bee venom allergic individuals was similar in magnitude and pattern to that observed with PLA2 and whole bee venom.

### Increased CD1a reactivity to wasp venom in allergic individuals

**Figure 3:**
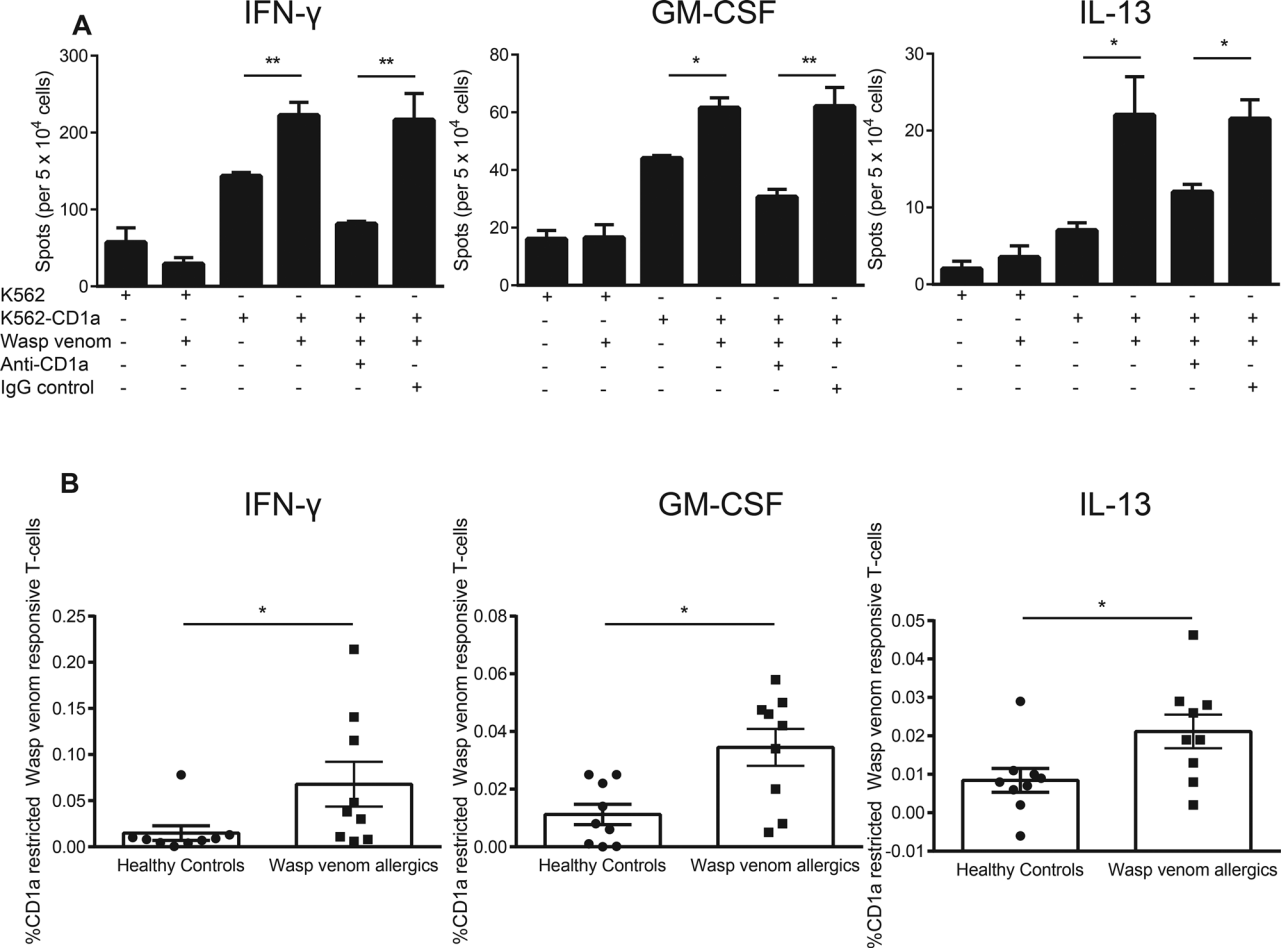
Wasp allergic individuals show increased levels of wasp venom responsive CD1a‐reactive T cells compared to nonallergic individuals. CD3+ T cells were isolated from peripheral blood of nonallergic and (*n* = 9) wasp allergic individuals (*n* = 9) by magnetic bead separation. (A) CD1a reactivity was examined by ELISpot with K562 or K562‐CD1a in the presence or absence of wasp venom (1 μg/mL) and/or 10 μg/mL anti‐CD1a mAb (OKT6). Data bars are shown as mean ± SEM and are from one allergic donor of nine studied. (B) Frequency of CD1a‐reactive T cells responsive to wasp venom above the auto‐reactive response. Data are shown as mean ± SEM and are pooled from 18 independent experiments, each performed in duplicate. *p < 0.05; **p < 0.01; unpaired nonparametric *t* test.

Separately, we also investigated human T‐cell responses to wasp venom and CD1a. Adult wasp allergic individuals with a history of anaphylaxis to wasp venom, and a positive skin prick test or raised wasp venom‐specific IgE antibodies were recruited. In the presence of wasp venom and K562‐CD1a, T‐cell responses were observed, which were not seen in the absence of CD1a expression, absence of venom or after treating with anti‐CD1a blocking antibody. Patterns of IFN‐γ, GM‐CSF, and IL‐13 were similar in detailed testing of one individual (Fig. 3A). As with bee venom allergy, increased in IFN‐γ (*p* < 0.05; Fig. 3B, left panel), GM‐CSF (*p* < 0.05; Fig. 3B, middle panel) and IL‐13 were all significant (*p* < 0.05; Fig. 3B, right panel) when comparing each of these cytokines between a cohort of wasp venom allergic and nonallergic individuals. Thus human CD1a‐mediated responses to bee and wasp venom are both elevated in allergic individuals and are similar in frequency, strength, and cytokine pattern.

### Cross‐reactivity of wasp and bee venom responsive CD1a‐ reactive T cells

**Figure 4:**
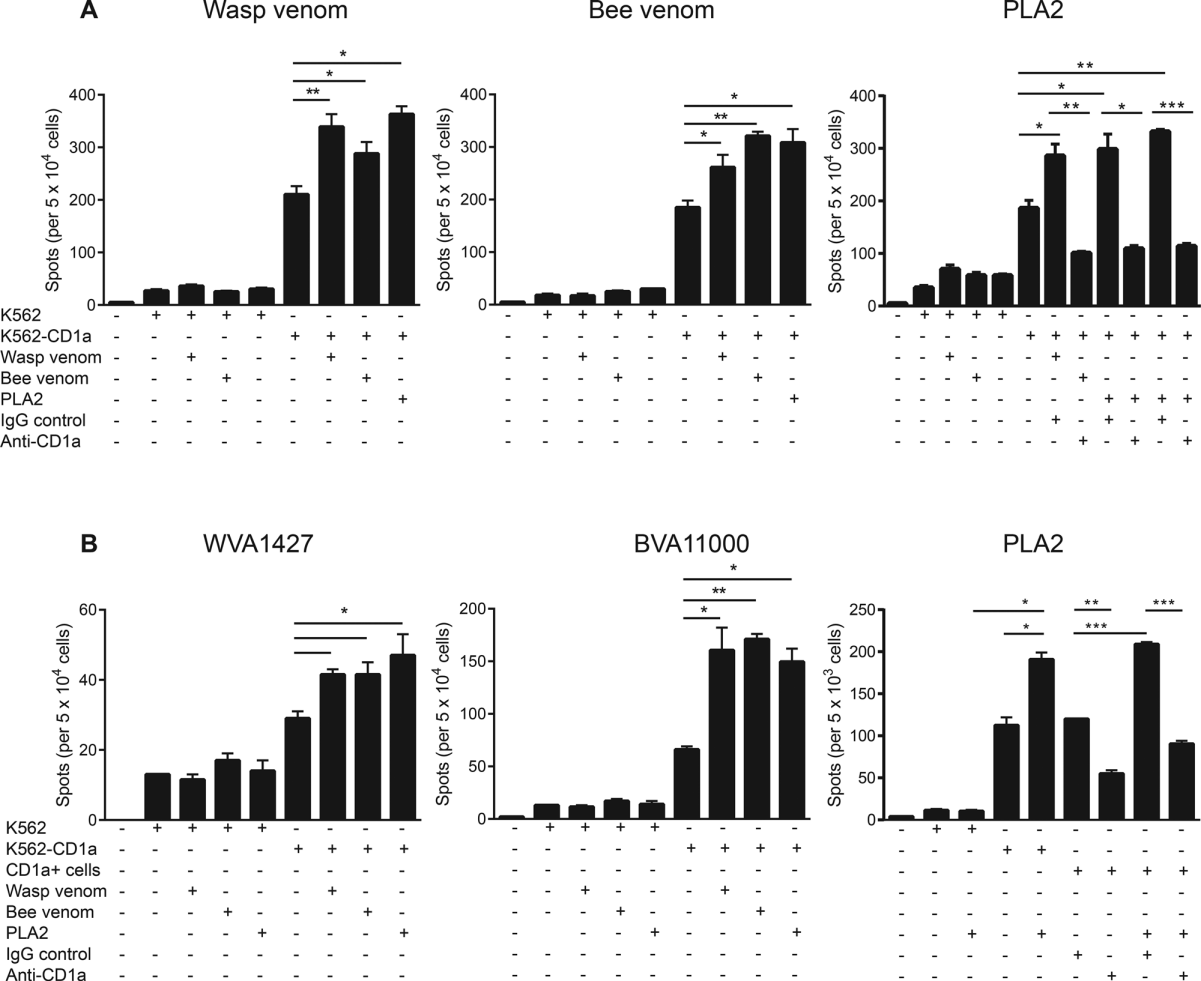
Cross‐venom CD1a‐reactive T‐cell responses. T‐cell lines were generated by culturing CD3+ T cells from adult human peripheral blood for 10–14 days in the presence of wasp venom‐pulsed, bee venom‐pulsed or bee PLA2‐pulsed irradiated K562‐CD1a cells. (A) CD1a reactivity of antigen enriched T‐cell lines from wasp venom allergic (left panel) or bee venom allergic (middle and right panels) individuals was examined by IFNγ‐ELISpot in response to K562‐CD1a cells in the presence of wasp venom, bee venom, or bee PLA2. Data bars are shown as mean ± SEM and are representative of three donors with wasp venom allergy and three donors with bee venom allergy, respectively. (B) CD1a reactivity of ex‐vivo T cells from a wasp (WVA; left panel) and a bee venom allergic (BVA; middle panel) individual was examined by IFN‐γ‐ELISpot in response to K562‐CD1a cells in the presence of wasp venom, bee venom, or bee PLA2. Skin‐derived CD1a‐positive cells were pulsed with bee PLA2 and incubated with a PLA2‐responsive CD1a‐reactive T‐cell line and IFN‐γ production was measured by ELISpot (right panel). Data bars are shown as mean ± SEM and are representative of three donors with wasp venom allergy and three donors with bee venom allergy, respectively. *p < 0.05; **p < 0.01; ***p < 0.001; unpaired nonparametric *t* test.

Prior studies completed outside the context of CD1a have shown that individual patients with allergy to bee or wasp show a predisposition to show cross‐sensitization and cross‐reactivity 26. To investigate whether wasp and bee allergic individuals respond to the same ligands presented by CD1a, we cultured CD3+ T cells from adult individuals with wasp‐venom pulsed, bee venom‐pulsed or bee PLA2‐pulsed and irradiated K562‐CD1a APCs for 10–14 days. The resulting T‐cell lines derived with one antigen were then tested for cross‐reactivity to all three antigens. T cells cultured in the presence of wasp venom and CD1a were enriched for wasp venom responsive CD1a‐reactive T cells. However, these cells were also able to respond to bee venom and bee PLA2. (Fig. 4A left panel). Similarly, T cells cultured in the presence of bee venom (Fig. 4A, middle panel) or PLA2 (Fig. 4A, right panel) and CD1a were also able to respond to wasp venom.

The simplest explanation is the T‐cell lines are responding to the same or similar antigens in each case. This favored interpretation is also supported by a prior study, which dissected the role of wasp and bee venom PLA 21. This study and other studies of the specificity of wasp and bee venom PLA, demonstrated that PLA acts to generate free fatty acids and lysophospholipids 21, both of which are known as antigens for CD1a or CD1d 15, 27. Last, it is notable that the absolute response to CD1a of human T‐cell lines generated by repeated exposure to CD1a is higher (Fig. 4), and in some cases more than tenfold higher than seen directly ex vivo (Fig. 1, 2, 3). This result is not expected if venoms are providing foreign lipid antigens but is expected if T cells are responding to self‐lipid antigens that are more efficiently liberated by wasp‐derived enzymes.

Separate studies of ex vivo polyclonal T‐cell responses among allergic individuals found that T cells from wasp venom allergic individuals (Fig. 4B left panel) responded to wasp venom, bee venom, and bee PLA2 and vice versa (Fig. 4B, middle panel). Having shown that bee PLA2 can reproduce the response seen to whole venom, this further suggests that wasp and bee venom responsive CD1a‐reactive T cells may respond to the same ligands, generated by PLA2. We have further demonstrated that the T‐cell responses to PLA2 are not restricted to the K562 cell line, but are also generated with primary skin‐derived CD1a+ cells (Fig. 4B right panel).

### Venom immunotherapy modulates CD1a‐ reactive T‐cell frequency and function

**Figure 5:**
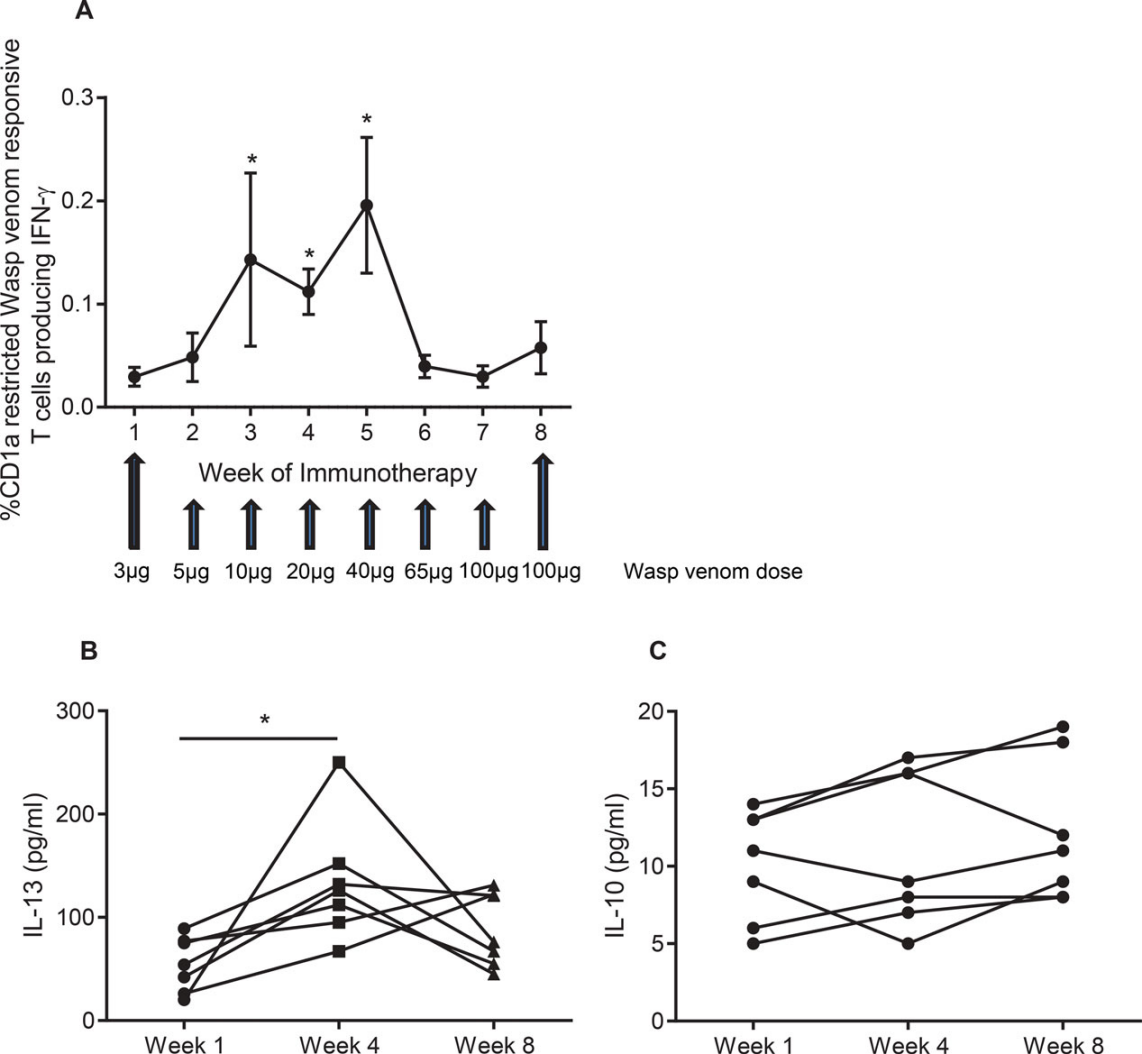
Longitudinal cytokine responses to immunotherapy in wasp venom allergic patients. CD3+ T cells were isolated from peripheral blood by magnetic bead separation from wasp venom allergic individuals, following polyclonal PBMC expansion. (A) Polyclonal wasp venom responsive CD1a‐reactive T‐cell frequencies were measured in nine wasp venom allergic individuals by IFN‐γ ELISpot with K562 or K562‐CD1a in the presence or absence of wasp venom (1 μg/mL). Data are shown as mean ± SEM and are pooled from nine independent experiments, each performed in duplicate. IL‐13 (B) and IL‐10 (C) secretion in response to wasp venom by CD1a‐reactive T cells from seven wasp venom allergic individuals were measured by ELISA. Data are shown as mean and are pooled from seven independent experiments, each performed in duplicate. *p < 0.05; Wilcoxon matched‐pairs signed rank test.

Subcutaneous immunotherapy is a highly effective treatment for patients with systemic allergic venom reactions 28. However, the underlying mechanisms are still not fully understood. Having shown that CD1a‐reactive T cells respond to venom‐derived antigens, we next investigated whether these cells were involved in clinical tolerance induction and desensitization. We analyzed T cells isolated from peripheral blood of nine wasp venom allergic individuals, taken at weekly intervals for 8 weeks, just prior to antigen administration. All patients achieved clinical tolerance to wasp venom by week 8, which was demonstrated as the ability to tolerate the equivalent of two sting doses (100 μg). We examined polyclonal T‐cell responses by ELISpot analysis of IFN‐γ release to K562 or K562‐CD1a in the presence or absence of wasp venom. Initially, with increasing allergen dose, we observed induction of IFN‐γ producing wasp venom responsive CD1a‐reactive T cells (*p* < 0.05), reaching a peak at week 5 of immunotherapy (Fig. 5A). However, the frequency of these cells then fell by week 6 and reached preimmunotherapy levels by week 8 (Fig. 5A), despite continued increases in antigen dose administered. We also measured IL‐13 and IL‐10 secretion in ELISpot supernatant by ELISA. A similar increase in IL‐13 levels was detected above the autoreactive response, in the presence of K562‐CD1a and wasp venom, peaking at week 4–5 (*p* < 0.05), and falling by week 8 (Fig. 5B), suggesting the loss of functional Th2 responses. IL‐10 secretion by wasp venom responsive CD1a‐reactive T cells did not significantly vary over the course of immunotherapy but the responses were close to the limit of detection, suggesting that IL‐10 is not produced in large amounts by the T cells (Fig. 5C). We have previously published data from the same cohort to show that the successful immunotherapy associated with wasp venom‐specific IgG4 production and induction of wasp venom peptide‐specific T cells at similar patterns to the lipid‐specific T cells presented herein 2. Overall these data demonstrate that CD1a‐reactive T‐cell responses to wasp venom are initially induced during immunotherapy, but IFN‐γ and IL‐13 responses are reduced toward the end of immunotherapy despite increasing antigen dose.

## Discussion

Venom allergy is an important cause of anaphylaxis, with a prevalence of 0.3–7.5% in the population 29 and has been studied as a model of hypersensitivity and inflammation. Venom protein‐ and peptide‐specific responses have been relatively well characterized in venom allergy 2, and allergic individuals are known to have raised levels of allergen‐specific Th2, B cells, and IgE 30, 31, with infiltration of allergen‐specific T cells in the skin and increased production of Th2 cytokines 31. Having shown previously that CD1a‐reactive T cells from blood and skin of healthy individuals respond to wasp and bee venom 21, here we show that venom allergic individuals have a higher frequency of venom responsive IFN‐γ, GM‐CSF, and IL‐13 producing CD1a‐reactive T cells. IL‐13 is associated with type 2 immune responses and allergic inflammation, particularly through the promotion of IgE class switching. CD1a‐reactive T‐cell production of IL‐13 might therefore promote venom peptide‐specific Th2 responses and subsequent IgE production. It is of interest that the CD1a‐reactive T cells were detectable without an antigen‐specific expansion step, in contrast to our studies of peptide‐specific Th2 responses from the same cohort 2. Although the specific cause for ex vivo detection in this case is not known, CD1a autoreactive T cells have a high precursor frequency in the skin 19. In the skin, IL‐13 upregulates keratinocyte expression of matrix metalloprotease (MMP) 9 and chemokine ligand (CCL) 22 32, 33. MMP is involved in tissue remodeling and so facilitates infiltrating cell migration 32. CCL22 acts as a chemoattractant for T cells expressing chemokine (C‐C motif) receptor (CCR) 4. CCR4 is predominantly expressed on Th2 cells 34 and is important for skin infiltration in disease 35. Indeed, IL‐13 stimulated keratinocytes were shown to preferentially attract CCR4+CD4+ cells 33.

GM‐CSF has a wide range of effector functions, altering T‐cell responses directly or indirectly, by influencing APC. In the skin, GM‐CSF is produced by and influences many cells, including enhancing keratinocyte growth and LC recruitment and maturation 36, 37. It can therefore act as a link between the innate and adaptive immune response, and so could contribute to the allergic inflammation in venom allergy. IFN‐γ is a multifunctional cytokine that is important in skin inflammation 38, 39. It is known to upregulate keratinocyte expression of human leukocyte antigen, cell adhesion proteins, and chemokines. This is further enhanced by Th2 cytokines IL‐13 and IL‐4 33, facilitating leukocyte infiltration, and peptide presentation, allowing IFN‐γ to further drive the allergic response.

Insects inject venom to kill prey, parasitize, or repel predators and so contain different toxic substances, including PLA. We have previously shown that venom PLA injected into the skin cleaves phospholipid substrates from the skin or from venom itself 21. Products of venom PLA activity, including free fatty acids and lysophospholipids, were recently shown to bind CD1a, found on nearby LCs and dermal dendritic cells 15, 16, 21 and indeed the skin is abundant in CD1a‐binding lipids 16. These neolipids generated by PLA activity are recognized by CD1a‐reactive T cells 21. Here, we have shown that PLA2 responsive CD1a‐reactive T‐cell responses are increased in bee venom allergic compared to nonallergic individuals. Through the initiation of IL‐13, GM‐CSF, IFN‐γ secretion, and potentially other cytokines, PLA2 may help drive the allergen peptide‐specific Th2 response and protein‐specific IgE response. Indeed elegant studies have shown that PLA‐derived peptides represent an important target for T cells from venom allergic individuals and can be used to modulate clinical reactivity 3, 4, 5.

CD1a expression is highly restricted to certain tissues, with the most abundant expression on LCs, and CD1a is expressed on more myeloid DC subsets that are migratory and often seen in the dermis 17, 18. Natural ligands for CD1a, such as wax esters, free fatty acids, and squalene are found in the stratum corneum, separating antigen presenting molecule and ligand under steady‐state conditions. Therefore, current models 19 emphasize local release of self‐antigens that can augment T‐cell responses to CD1a, suggesting that venom injected into the skin triggers the cleavage of intact self‐phospholipids into activating forms. The identity of antigens was solved in other studies 15, 16, 21 but our current results do show that treatment of T cells with active venoms increases the observed CD1a reactivity.

In considering the cross‐reactivity among insect species, we found previously that in addition to having its well‐known PLA1 activity, wasp venom PLA also has a degree of PLA2 activity so that the two venoms would be expected to have similar effects on host phospholipids 21. Here, we show that CD1a‐reactive T cells responding to wasp venom can also recognize bee venom and vice versa. This further suggests that wasp and bee venom PLA may act on similar substrates and generate similar CD1a reactive lipid antigens, resulting in a cross‐venom CD1a‐reactive T‐cell response. Indeed it is known that some individuals with bee venom allergy are also allergic to wasp venom suggesting that common pathways of allergenicity may be involved 26. This raises the possibility that future diagnostic or immunotherapeutic approaches may target the shared CD1a‐reactive response to modulate the subsequent allergic process and provides a specific rationale to consider PLA and the lipid products of PLA, namely fatty acids and lysolipids, as immunodiagnostic and immunotherapeutic targets. Having established raised frequencies of wasp venom responsive CD1a‐reactive T cells in venom allergy, further studies could focus on their influence on the peptide‐specific immune response, including allergen‐specific B cells and IgE production.

Immunotherapy provides an opportunity to understand the mechanisms involved in generating and maintaining long‐term clinical tolerance. This is likely to be important in the future treatment of a number of immune disorders, including allergy and autoimmunity 40, 41. Within days of commencing immunotherapy, a decrease in mast cell and basophil activation and degranulation has been reported 42, 43. Allergen‐specific effector T and B cells shifting toward a regulatory phenotype appears to be important, inducing effector T‐cell tolerance, decreasing proinflammatory cytokines from mast cells, decreasing eosinophil function and activity, and directing allergen‐specific antibody isotype from IgE to IgG4 9, 28. We show here the induction of wasp venom responsive CD1a‐reactive T cells producing IFN‐γ and IL‐13 during the first 4 weeks of immunotherapy, providing further evidence that CD1a‐reactive T cells respond to wasp venom and are generated through antigen exposure. However, we show a reduction in IFN‐γ and IL‐13 producing CD1a‐reactive T cells in response to wasp venom from week 4 of immunotherapy despite increasing antigen dose. Whether this reduction in IFN‐γ and IL‐13 producing CD1a‐reactive T cells is sustained beyond week 8 is unknown, however, it is noteworthy that it coincides with the induction of clinical tolerance, suggesting that wasp venom responsive CD1a‐reactive T cells may play a role in allergic disease. It is possible that wasp venom responsive CD1a‐reactive T cells become less responsive or even anergic, with continued exposure to increasing doses of allergen.

It is interesting to note that during a sting, venom is delivered directly into the epidermis and dermis where CD1a‐expressing cells are present. In contrast, during immunotherapy, venom is delivered subcutaneously, where there are fewer CD1a‐expressing cells. This leads to the prediction that successful immunotherapy may partly depend on site of delivery. This is indeed the case, as subcutaneous or oral delivery of grass pollen lead to systemic clinical tolerance. It is also of note that peptide‐based immunotherapy can produce clinical efficacy, consistent with the possibility that it is the lipids that contribute the allergic process 5. Although we observed an increase in frequency of venom responsive T cells in the first few weeks of immunotherapy, we speculate that immunotherapy achieves clinical tolerance in part by bypassing CD1a and therefore modulating the phenotype of the subsequent peptide‐specific T‐cell and IgE responses.

In summary, we have shown increased frequency of venom responsive CD1a‐retricted T cells in allergic individuals, suggesting a role for these cells in allergic disease. The loss of responses seen during immunotherapy despite increasing antigen doses, suggests that CD1a‐reactive responses may shape the subsequent peptide‐specific and IgE responses. The findings have implications for mechanisms of allergy and of clinical tolerance induction, with relevance to many forms of hypersensitivity. Whereas all current approaches focus on the role of proteins or peptides in T‐cell response, these findings support a broad new view in which CD1a and nonpeptide antigens could participate in allergy. Because many known allergens are nonspecific small molecules, the proof of principle for a role of CD1a in allergic responses could promote generally new approaches to immunodiagnosis and treatment.

## Materials and methods

### Donors

PBMC were isolated from healthy adult donors (age range 25–50 years) with a previous history of bee or wasp sting, and from wasp and bee allergic individuals with a history of anaphylaxis under local ethics approval (CO2.291 and 09/H0606/71). T cells were isolated using CD3 MACs bead separation (Miltenyi Biotec, Germany) for ex‐vivo assays. Or, PBMCs were expanded by culturing with feeder cells for 14–20 days (1000 patient PBMCs plus 0.2 × 10^6^ irradiated donor PBMCs and 0.4 × 10^6^ transformed B‐cell line per well of a round‐bottom 96‐well plate in RPMI supplemented with 100 IU/mL penicillin, 100 μg/mL streptomycin, 2 mM L‐glutamine, and 5% human serum (Sigma‐Aldrich, MO, USA), nonessential amino acids, HEPES, sodium pyruvate, and 2‐mercaptoethanol (Life Technologies, CA, USA) in the presence of 1 nM IL‐2 (PeproTech, NJ, USA) and 50 ng/mL anti‐CD3 antibody (OKT3). After cell numbers had expanded, T cells were isolated using CD3 MACs beads (Miltenyi Biotec, Germany). T‐cell lines were generated using CD3+ cell isolated by MACs bead separation from healthy adult human peripheral blood. T cells were then cultured with wasp venom, bee venom, or PLA2 pulsed irradiated K562‐CD1a cells for 10–14 days. T‐cell lines were further enriched for PLA2 reactivity using the IFN‐γ secretion assay (Miltenyi Biotec, Germany) following manufacturer instructions.

### Isolation of CD1a+ cells

Migratory, adherent cells from the epidermis of skin sections were harvested following 5 days of culture in complete media and enriched for CD1a^+^ cells by MACs cell separation (Miltenyi Biotec) 21. The CD1a^+^ cells were incubated with 10 μg/mL anti‐HLA‐ABC and anti‐HLA‐DR blocking antibodies (W6/32 and L243, respectively, for 1 h before coculture with T cells, to minimize HLA‐restricted responses.

### ELISpot

CD1a reactivity was assessed by IFN‐γ, GM‐CSF, and IL‐13 ELISPOT (Mabtech AB, Sweden). ELISpot plates (Millipore Corp., MA, USA) were coated with anti‐cytokine antibody overnight (Mabtech AB, Sweden). K562 cells were pulsed with 1 μg/mL venom or PLA2 (Sigma‐Aldrich) overnight, and were then washed and resuspended in R5* (RPMI supplemented with 2 mM L‐glutamine, 100I U/mL penicillin, and 100 μg/mL streptomycin plus 5% human serum). The plates were washed six times with RPMI and blocked for 1 h with RPMI supplemented with 2 mM L‐glutamine, 100 IU/mL penicillin, and 100 μg/mL streptomycin plus 10% human serum (R10*). A total of 50 000 T cells were added per well to which 25 000 K562 cells were added. In some experiments 10 μg/mL anti‐CD1a blocking antibody (OKT‐6) or 10 μg/mL IgG1 isotype control were added to K562 before addition of T cells. Wells were set‐up in duplicate or triplicate. Phorbol myristate acetate 10 ng/mL and Ionomycin 500 ng/mL was included as a positive control, and T cells alone in the absence of K562 was included as a negative control. After overnight incubation at 37°C and 5% CO_2_, culture supernatants were recovered, and plates were washed x 6 in PBS‐Tween 0.05% and incubated with 1 μg/mL of biotin‐linked anti‐IFN‐γ monoclonal antibody (Mabtech AB, Sweden) for 2 h. After washing x 6 in PBS‐Tween 0.05%, the plates were incubated for a further 1 h with streptavidin‐alkaline phosphatase (Mabtech AB, Sweden). Spots were visualized using an alkaline phosphatase conjugate substrate kit (Biorad, Hercules, CA, USA) and enumerated using an automated ELISpot reader (Autimmun Diagnostika gmbh ELISpot Reader Classic, Germany).

### ELISA

IL‐10 and IL‐13 production was measured in recovered ELISpot supernatants by ELISA (Human IL‐10 Ready‐Set‐Go!^®^; Ebioscience CA, USA (2–300 pg/mL range), and IL‐13 Human Ultrasensitive ELISA Kit; Life Technologies, CA, USA (0.78–50 pg/mL range)), as per manufacturers’ instructions.

### Statistics

Allergic and healthy donors investigated for CD1a‐reactive wasp venom, bee venom, and PLA2‐specific responses were analyzed using one‐tailed Mann–Whitney unpaired *t* tests. Changes detected over the course of immunotherapy were analyzed using one‐tailed Wilcoxon matched‐pairs signed rank test.

## Conflict of interest

G.O. has served on Novartis Advisory Boards. All other contributing authors confirm that there are no commercial affiliations, stock, or equity interests, or patent‐licensing arrangements that could be considered to pose a financial conflict of interest.

**Abbreviations**
CCL: chemokine ligand
CCR: chemokine (C‐C motif) receptor
LC: Langerhans cell
MMP: matrix metalloprotease
PLA: phospholipase A

## Supporting information

As a service to our authors and readers, this journal provides supporting information supplied by the authors. Such materials are peer reviewed and may be re‐organized for online delivery, but are not copy‐edited or typeset. Technical support issues arising from supporting information (other than missing files) should be addressed to the authors.

Peer Review Correspondence

## References

[1] Biló, B. M. , Rueff, F. , Mosbech, H. , Bonifazi, F. and Oude‐Elberink, J. N. G. , Diagnosis of hymenoptera venom allergy. Allergy 2005 60: 1339–1349. 16197464 10.1111/j.1398-9995.2005.00963.x

[2] Aslam, A. , Chan, H. , Warrell, D. A. , Misbah, S. and Ogg, G. S. , Tracking antigen‐specific T‐cells during clinical tolerance induction in humans. PloS One 2010 5: e11028. 20543955 10.1371/journal.pone.0011028 PMC2882953

[3] Akdis, C. A. , Akdis, M. , Blesken, T. , Wymann, D. , Alkan, S. S. , Müller, U. and Blaser, K. , Epitope‐specific T cell tolerance to phospholipase A2 in bee venom immunotherapy and recovery by IL‐2 and IL‐15 in vitro. J. Clin. Invest. 1996 98: 1676. 8833918 10.1172/JCI118963 PMC507602

[4] Akdis, C. A. , Blesken, T. , Akdis, M. , Alkan, S. S. , Wüthrich, B. , Heusser, C. H. and Blaser, K. , Induction and differential regulation of bee venom phospholipase A 2–specific human IgE and IgG 4 antibodies in vitro requires allergen‐specific and nonspecific activation of T and B cells. J. Allergy Clin. Immunol. 1997 99: 345–353. 9058690 10.1016/s0091-6749(97)70052-6

[5] Müller, U. , Akdis, C. A. , Fricker, M. , Akdis, M. , Blesken, T. , Bettens, F. and Blaser, K. , Successful immunotherapy with T‐cell epitope peptides of bee venom phospholipase A2 induces specific T‐cell anergy in patients allergic to bee venom. J. Allergy Clin. Immunol. 1998 101: 747–754. 9648701 10.1016/S0091-6749(98)70402-6

[6] Wachholz, P. A. and Durham, S. R. , Mechanisms of immunotherapy: IgG revisited. Curr. Opin. Allergy Clin. Immunol. 2004 4: 313–318. 15238798 10.1097/01.all.0000136753.35948.c0

[7] Wambre, E. , DeLong, J. H. , James, E. A. , Torres‐Chinn, N. , Pfützner, W. , Möbs, C. , Durham, S. R. **et al.,** Specific immunotherapy modifies allergen‐specific CD4+ T‐cell responses in an epitope‐dependent manner. J. Allergy Clin. Immunol. 2014 133: 872–879.e7. 24373351 10.1016/j.jaci.2013.10.054 PMC3961577

[8] Akdis, C. A. and Akdis, M. , Mechanisms of allergen‐specific immunotherapy. J. Allergy Clin. Immunol. 2011 127: 18–27. 21211639 10.1016/j.jaci.2010.11.030

[9] Akdis, M. and Akdis, C. A. , Mechanisms of allergen‐specific immunotherapy: multiple suppressor factors at work in immune tolerance to allergens. J. Allergy Clin. Immunol. 2014 133: 621–631. 24581429 10.1016/j.jaci.2013.12.1088

[10] Mori, L. and De Libero, G. , Presentation of lipid antigens to T cells. Immunol. Lett. 2008 117: 1–8. 18243339 10.1016/j.imlet.2007.11.027

[11] Gumperz, J. E. , The ins and outs of CD1 molecules: bringing lipids under immunological surveillance. Traffic 2006 7: 2–13. 16445682 10.1111/j.1600-0854.2005.00364.x

[12] Layre, E. , de Jong, A. and Moody, D. B. , Human T cells use CD1 and MR1 to recognize lipids and small molecules. Curr. Opin. Chem. Biol. 2014 23: 31–38. 25271021 10.1016/j.cbpa.2014.09.007

[13] Zajonc, D. M. , Crispin, M. D. , Bowden, T. A. , Young, D. C. , Cheng, T. Y. , Hu, J. , Costello, C. E. **et al.,** Molecular mechanism of lipopeptide presentation by CD1a. Immunity 2005 22: 209–219. 15723809 10.1016/j.immuni.2004.12.009

[14] Zajonc, D. M. , Elsliger, M. A. , Teyton, L. and Wilson, I. A. , Crystal structure of CD1a in complex with a sulfatide self‐antigen at a resolution of 2.15 A. Nat. Immunol. 2003 4: 808–815. 12833155 10.1038/ni948

[15] Birkinshaw, R. W. , Pellicci, D. G. , Cheng, T. Y. , Keller, A. N. , Sandoval‐Romero, M. , Gras, S. , de Jong, A. **et al.,** Alphabeta T cell antigen receptor recognition of CD1a presenting self‐lipid ligands. Nat. Immunol. 2015 16: 258–266. 25642819 10.1038/ni.3098 PMC7103088

[16] de Jong, A. , Cheng, T. Y. , Huang, S. , Gras, S. , Birkinshaw, R. W. , Kasmar, A. G. , Van Rhijn, I. **et al.,** CD1a‐autoreactive T cells recognize natural skin oils that function as headless antigens. Nat. Immunol. 2014 15: 177–185. 24362891 10.1038/ni.2790 PMC3932764

[17] Yakimchuk, K. , Roura‐Mir, C. , Magalhaes, K. G. , de Jong, A. , Kasmar, A. G. , Granter, S. R. , Budd, R. **et al.,** *Borrelia burgdorferi* infection regulates CD1 expression in human cells and tissues via IL1‐beta. Eur. J. Immunol. 2011 41: 694–705. 21246541 10.1002/eji.201040808 PMC3082368

[18] Dougan, S. K. , Kaser, A. and Blumberg, R. S. , CD1 expression on antigen‐presenting cells. Curr. Topics Microbiol. Immunol. 2007 314: 113–141. 10.1007/978-3-540-69511-0_5 17593659

[19] de Jong, A. , Pena‐Cruz, V. , Cheng, T. Y. , Clark, R. A. , Van Rhijn, I. and Moody, D. B. , CD1a‐autoreactive T cells are a normal component of the human alphabeta T cell repertoire. Nat. Immunol. 2010 11: 1102–1109. 21037579 10.1038/ni.1956 PMC3131223

[20] de Lalla, C. , Lepore, M. , Piccolo, F. M. , Rinaldi, A. , Scelfo, A. , Garavaglia, C. , Mori, L. **et al.,** High‐frequency and adaptive‐like dynamics of human CD1 self‐reactive T cells. Eur. J. Immunol. 2011 41: 602–610. 21246542 10.1002/eji.201041211

[21] Bourgeois, E. A. , Subramaniam, S. , Cheng, T.‐Y. , De Jong, A. , Layre, E. , Ly, D. , Salimi, M. **et al.,** Bee venom processes human skin lipids for presentation by CD1a. J. Exp. Med. 2015 212: 149–163. 25584012 10.1084/jem.20141505 PMC4322046

[22] Seino, K. i. , Motohashi, S. , Fujisawa, T. , Nakayama, T. and Taniguchi, M. , Natural killer T cell‐mediated antitumor immune responses and their clinical applications. Cancer Sci. 2006 97: 807–812. 16805854 10.1111/j.1349-7006.2006.00257.x PMC11158813

[23] Brigl, M. and Brenner, M. B. , CD1: antigen presentation and T cell function. Ann. Rev. Immunol. 2004 22: 817–890. 15032598 10.1146/annurev.immunol.22.012703.104608

[24] Akbari, O. , Stock, P. , Meyer, E. , Kronenberg, M. , Sidobre, S. , Nakayama, T. , Taniguchi, M. **et al.,** Essential role of NKT cells producing IL‐4 and IL‐13 in the development of allergen‐induced airway hyperreactivity. Nat. Med. 2003 9: 582–588. 12669034 10.1038/nm851

[25] Agea, E. , Russano, A. , Bistoni, O. , Mannucci, R. , Nicoletti, I. , Corazzi, L. , Postle, A. D. **et al.,** Human CD1‐restricted T cell recognition of lipids from pollens. J. Exp. Med. 2005 202: 295–308. 16009719 10.1084/jem.20050773 PMC2213012

[26] Müller, U. , Schmid‐Grendelmeier, P. , Hausmann, O. and Helbling, A. , IgE to recombinant allergens Api m 1, Ves v 1, and Ves v 5 distinguish double sensitization from crossreaction in venom allergy. Allergy 2012 67: 1069–1073. 22676144 10.1111/j.1398-9995.2012.02847.x

[27] Fox, L. M. , Cox, D. G. , Lockridge, J. L. , Wang, X. , Chen, X. , Scharf, L. , Trott, D. L. **et al.,** Recognition of lyso‐phospholipids by human natural killer T lymphocytes. PLoS Biology 2009 7: e1000228. 19859526 10.1371/journal.pbio.1000228 PMC2760207

[28] Ozdemir, C. , Kucuksezer, U. , Akdis, M. and Akdis, C. , Mechanisms of immunotherapy to wasp and bee venom. Clin. Exp. Allergy 2011 41: 1226–1234. 21729181 10.1111/j.1365-2222.2011.03812.x

[29] Krishna, M. , Ewan, P. , Diwakar, L. , Durham, S. , Frew, A. , Leech, S. and Nasser, S. , Diagnosis and management of hymenoptera venom allergy: British Society for Allergy and Clinical Immunology (BSACI) guidelines. Clin. Exp. Allergy 2011 41: 1201–1220. 21848758 10.1111/j.1365-2222.2011.03788.x

[30] Romagnani, S. , The role of lymphocytes in allergic disease. J. Allergy Clin. Immunol. 2000 105: 399–408. 10719286 10.1067/mai.2000.104575

[31] Maggi, E. , T cell responses induced by allergen‐specific immunotherapy. Clin. Exp. Immunol. 2010 161: 10–18. 20408857 10.1111/j.1365-2249.2010.04148.x PMC2940143

[32] Purwar, R. , Kraus, M. , Werfel, T. and Wittmann, M. , Modulation of keratinocyte‐derived MMP‐9 by IL‐13: a possible role for the pathogenesis of epidermal inflammation. J. Invest. Dermatol. 2008 128: 59–66. 17597813 10.1038/sj.jid.5700940

[33] Purwar, R. , Werfel, T. and Wittmann, M. , IL‐13‐stimulated human keratinocytes preferentially attract CD4+ CCR4+ T cells: possible role in atopic dermatitis. J. Invest. Dermatol. 2006 126: 1043–1051. 16484990 10.1038/sj.jid.5700085

[34] Bonecchi, R. , Bianchi, G. , Bordignon, P. P. , D'Ambrosio, D. , Lang, R. , Borsatti, A. , Sozzani, S. **et al.,** Differential expression of chemokine receptors and chemotactic responsiveness of type 1 T helper cells (Th1s) and Th2s. J. Exp. Med. 1998 187: 129–134. 9419219 10.1084/jem.187.1.129 PMC2199181

[35] Wakugawa, M. , Hayashi, K. , Nakamura, K. and Tamaki, K. , Evaluation of mite allergen‐induced Th1 and Th2 cytokine secretion of peripheral blood mononuclear cells from atopic dermatitis patients: association between IL‐13 and mite‐specific IgE levels. J. Dermatol. Sci. 2001 25: 116–126. 11164708 10.1016/s0923-1811(00)00118-3

[36] Heufler, C. , Koch, F. and Schuler, G. , Granulocyte/macrophage colony‐stimulating factor and interleukin 1 mediate the maturation of murine epidermal Langerhans cells into potent immunostimulatory dendritic cells. J. Exp. Med. 1988 167: 700–705. 3279156 10.1084/jem.167.2.700 PMC2188828

[37] Kaplan, G. , Walsh, G. , Guido, L. , Meyn, P. , Burkhardt, R. , Abalos, R. , Barker, J. **et al.,** Novel responses of human skin to intradermal recombinant granulocyte/macrophage‐colony‐stimulating factor: Langerhans cell recruitment, keratinocyte growth, and enhanced wound healing. J. Exp. Med. 1992 175: 1717–1728. 1588289 10.1084/jem.175.6.1717 PMC2119267

[38] Grine, L. , Dejager, L. , Libert, C. and Vandenbroucke, R. E. , An inflammatory triangle in psoriasis: TNF, type I IFNs and IL‐17. Cytokine Growth Factor Rev. 2014 26: 25–33. 25434285 10.1016/j.cytogfr.2014.10.009

[39] Zaidi, M. R. and Merlino, G. , The two faces of interferon‐γ in cancer. Clin. Cancer Res. 2011 17: 6118–6124. 21705455 10.1158/1078-0432.CCR-11-0482 PMC3186825

[40] Xia, W. , Wang, J. , Xu, Y. , Jiang, F. and Xu, L. , L‐BLP25 as a peptide vaccine therapy in non‐small cell lung cancer: a review. J. Thoracic Dis. 2014 6: 1513. 10.3978/j.issn.2072-1439.2014.08.17 PMC4215136 25364531

[41] Sabatos‐Peyton, C. A. , Verhagen, J. and Wraith, D. C. , Antigen‐specific immunotherapy of autoimmune and allergic diseases. Curr. Opin. Immunol. 2010 22: 609–615. 20850958 10.1016/j.coi.2010.08.006 PMC2977065

[42] Novak, N. , Mete, N. , Bussmann, C. , Maintz, L. , Bieber, T. , Akdis, M. , Zumkehr, J. **et al.,** Early suppression of basophil activation during allergen‐specific immunotherapy by histamine receptor 2. J. Allergy Clin. Immunol. 2012 130: 1153–1158. e2. 22698521 10.1016/j.jaci.2012.04.039

[43] Eberlein‐Konig, B. , Ullmann, S. , Thomas, P. and Przybilla, B. , Tryptase and histamine release due to a sting challenge in bee venom allergic patients treated successfully or unsuccessfully with hyposensitization. Clin. Exp. Allergy 1995 25: 704–712. 7584681 10.1111/j.1365-2222.1995.tb00007.x

