# Supplementary material for: Elevated and cross‐responsive CD1a‐reactive T cells in bee and wasp venom allergic individuals
Source: Eur J Immunol. 2015 Nov 17;46(1):242–52. doi: 10.1002/eji.201545869 (PMC4738458; doi:10.1002/eji.201545869)
Supplement: Supplementary file 1 — Peer Review Correspondence [file EJI-46-242-s001.pdf]

European Journal of Immunology

Supporting Information
for
DOI 10.1002/eji.201545869

Sumithra Subramaniam, Aamir Aslam, Siraj A. Misbah, Mariolina Salio, Vincenzo Cerundolo, D Branch Moody and Graham Ogg

Elevated and cross-responsive CD1a-reactive T cells in bee and wasp venom allergic individuals

© 2015 The Authors. *European Journal of Immunology* published by WILEY-VCH Verlag GmbH & Co. KGaA, Weinheim.

European Journal of Immunology

Elevated and cross-responsive CD1a-reactive T cells in bee and wasp venom allergic individuals

Sumithra Subramaniam, Aamir Aslam, Siraj A.Misbah, Mariolina Salio, Vincenzo Cerundolo, D Branch Moody, Graham Ogg

Correspondence: Professor Graham Ogg, WIMM, JR Hospital, University of Oxford, Headley Way, Oxford, Oxfordshire OX3 9DS, United Kingdom

---

Review Timeline:	Submission date:	18-Jun-2015
	First Editorial decision:	14-Jul-2015
	First Revision received:	28-Aug-2015
	Accepted:	16-Oct-2015

Handling Executive Committee member: Prof. Maria Yazdanbakhsh

Please note that the correspondence below does not include the standard editorial instructions regarding preparation and submission of revised manuscripts, only the scientific revisions requested and addressed.

First Editorial Decision - 14-Jul-2015

Dear Mrs. Subramaniam,

Manuscript ID eji.201545869 entitled "Elevated and cross-responsive CD1a-reactive T cells in bee and wasp venom allergic individuals" which you submitted to the European Journal of Immunology has been reviewed. The comments of the referees are included at the bottom of this letter.

A revised version of your manuscript that takes into account the comments of the referees will be reconsidered for publication. Should you disagree with any of the referees' concerns, you should address this in your point-by-point response and provide solid scientific reasons for why you will not make the requested changes.

You should also pay close attention to the editorial comments included below. **In particular, please edit your figure legends to follow Journal standards as outlined in the editorial comments. Failure to do this will result in delays in the re-review process.**

Please note that submitting a revision of your manuscript does not guarantee eventual acceptance, and that your revision will be re-reviewed by the referees before a decision is rendered.

If the revision of the paper is expected to take more than three months, please inform the editorial office. Revisions taking longer than six months may be assessed by new referees to ensure the relevance and timeliness of the data.

Once again, thank you for submitting your manuscript to European Journal of Immunology and we look forward to receiving your revision.

Yours sincerely,
Katharina Schmidt

On behalf of
Prof. Maria Yazdanbakhsh

Dr. Katharina Schmidt
Editorial Office
European Journal of Immunology
e-mail: ejied@wiley.com
****

Reviewer: 1

Comments to the Author
In the manuscript entitled “Elevated and cross-responsive CD1a-reactive T cells in bee and wasp venom allergic individuals”• by Sumithra Subramaniam et al., they extend their previously published data on the CD1a-resticted recognition of bee and wasp venom components by T cells. Here they show that the responses are enhanced in allergic patients compared to healthy individuals.
This observation is in itself to be expected as this has also been demonstrated many times for MHC-II-restricted T cell responses. In addition, they show that PLA2 is the insulting component in bee venoms, which is also already published in their previous paper in J. Exp. Med. Moreover, there are some flaws in the experiments.

1. In figure 1 the reaction of peripheral blood T cells to bee venom. Compared to the CD1a- K562 cell line the response to the CD1a+ K562 cell line is already strongly increased, from approx. 10 to 40 spots,

although this increase is not significant. How do the authors explain this? In the text the authors mention presentation of K562-derived lipids but then they should show the same response while blocking CD1. More alarming is the fact that the bee venom response is not significantly enhanced. Only in the presence of IgG control vs anti-CD1a a statistically significant difference is observed. The same holds true for Fig 2 and Fig 3.

2. To really appreciate the data, Figure 1B should be shown by individual dots for each patients or control. Moreover, why are only 8 out of 13 patients shown in Figure 1B. Al this also holds true for Fig 2 and 3.

3. Data of Figure 1, 2 and 3 could be combined in 1 figure. Same message.

4. In figure 4 the authors show that wasp- and bee-specific T cells are cross-reactive using T cell lines. But what is the in vivo relevance of a barely 2-fold increase in reactivity? To what extent are MHC-II-restricted wasp- or bee-specific T cells cross-reactive?

5. Why did the authors measure during IT the response for IFNg by ELISPOT and IL-13 and IL-10 production in supernatant? Choosing either one of the methods would allow for a IFN-g/IL-4 ratio that you expect to see changing in favor of IFNg during IT. No wit shows that T cell reactivity is enhance during the middle part of IT with an increase in both IFNg and IL-13 without any effect on IL-10. The latter is on the detection limit of the ELISA, I guess, so does not add anything.

6. In the discussion the authors explain the functions of IFNg, IL-13 and GM-CSF but how do the levels of cytokine production compare to the production of these cytokines by MHC-II restricted responses. Do these amounts contribute?

7. In several parts of the manuscripts the authors state that CD1a is most abundantly expressed in the epidermis of the skin. However, there is also a substantial population of CD1a+ dermal DCs, and CD1a expression is also present on DC is in the respiratory tract. The numbers of CD1a+ LCs is always lower the CD1a+ DDCs and in all crawl-out skin models. This remark should be placed with more caution.

8. What is the nature of the K562 cell line that is used? Can this cell line process antigens like dendritic cells or are fragments presented in the CD1a molecules? Is this presentation also happening with skin-derived CD1a+ dendritic cells? Not monocyte-derived DCs as it is now clear that many processes are not as potent with ex vivo DCs compare to the Mo-DC models.

Reviewer: 2

Comments to the Author

This manuscript by Subramanian et al. shows that venom-responsive CD1a-reactive T cells are increased in the blood of venom allergic individuals. These cells are cross-reactive between bee and wasp-derived venoms, probably through the generation of similar self-lipid antigens by the phospholipase A2 activity of

both venoms. Furthermore, the authors show that following subcutaneous immunotherapy with increasing amount of venom, the frequency of venom-responsive CD1a-reactive T cells diminishes. These are new and interesting data that have implications for allergy and immunotherapy approaches. The experiments are properly performed and presented.

Comments: In Fig 4, it would important to show that the cross-reactive responses can be blocked specifically by the addition of anti-CD1a mAbs.

Reviewer: 3

Comments to the Author
This work describes T cell responses to neolipid antigens generated by bee and wasp venom phospholipase in non-allergic and allergic individuals as well as patients treated with allergen-specific immunotherapy. The approach is technically sound and the paper is concise and well written. The conclusions are supported by the data presented.

How did the authors evaluate the induction of tolerance in their patients, coinciding with the effects on CD1a-reactive T cells? (lines 292). It should be demonstrated that the patients in this study responded to treatment by tolerance induction.

Minor comments:
The information given in the discussion (lines 243-249) on the formation of neolipids by PLA should already be given in the introduction.

The IL-10 levels measured in cells from treated patients are very low, possibly below the limit of detection. What is the sensitivity limit of the applied assays?

<u>First Revision – authors' response - 28-Aug-2015</u>

Reviewer #1: Sumithra Subramaniam et al. extend their previously published data on the CD1a-resticted recognition of bee and wasp venom components by T cells. Here they show that the responses are enhanced in allergic patients compared to healthy individuals. This observation is in itself to be expected as this has also been demonstrated many times for MHC-II-restricted T cell responses. In addition, they show that PLA2 is the insulting component in bee venoms, which is also already published in their previous paper

in J. Exp. Med.

The referee correctly points to the discovery of CD1a mediated responses to venom earlier this year. Venoms cause an inflammatory response in nearly everyone but a dangerous hypersensitivity response in a small subset of humans. No aspect of the prior data in any way address the role of CD1a in the hypersensitivity response, so this is an entirely open and unexplored question that can be answered only with a study of this type. Just as the reviewer says, MHC II is highlighted on nearly every review or textbook on the subject but CD1a is highlighted in no review of the subject. This paper would be the first to directly implicate CD1a in hypersensitivity.

Moreover, there are some flaws in the experiments.

1. In figure 1 the reaction of peripheral blood T cells to bee venom. Compared to the CD1a- K562 cell line the response to the CD1a+ K562 cell line is already strongly increased, from approx. 10 to 40 spots, although this increase is not significant. How do the authors explain this? In the text the authors mention presentation of K562-derived lipids but then they should show the same response while blocking CD1. More alarming is the fact that the bee venom response is not significantly enhanced. Only in the presence of IgG control vs anti-CD1a a statistically significant difference is observed. The same holds true for Fig 2 and Fig 3.

Although statistical information was only provided comparing anti-CD1a vs isotype in the presence of venom as that was the key focus of the message, actually the anti-CD1a did also significantly inhibit the autoreactive response. We did not include this (and other statistical comparisons) in an attempt to make the figures easier to follow. However we agree that inhibiting the autoreactive response is also important to show, and so have added data to figure 1 to address this and also added other relevant statistical comparisons to figures 2 and 3 as suggested by the reviewer. Inhibition of the autoreactive T cell response by anti-CD1a would be compatible with presentation of K562 derived lipids by CD1a as has been reported previously (de Jong et al 2010 Nat Immunology; De Jong et al 2014 Nat Immunology).

2. To really appreciate the data, Figure 1B should be shown by individual dots for each patients or control. Moreover, why are only 8 out of 13 patients shown in Figure 1B. Al this also holds true for Fig 2 and 3.

Of the 13 individuals studied in Figure 1B, 8 were healthy controls and 5 were bee venom allergic individuals. The same principle applies for Fig 2 and 3. We have also revised the plots to include individual dots.

3. Data of Figure 1, 2 and 3 could be combined in 1 figure. Same message.

We believe the information is easier to follow as separate figures, as large amounts of data are presented. We would welcome your advice as to whether it would be better to combine them.

4. In figure 4 the authors show that wasp- and bee-specific T cells are cross-reactive using T cell lines. But what is the in vivo relevance of a barely 2-fold increase in reactivity? To what extent are MHC-II-restricted wasp- or bee-specific T cells cross-reactive?

Although only 2-fold increase in reactivity seen, this does correlate with disease, indicating they may be involved and indeed an in vivo effect in the skin may be greater. It has previously been demonstrated that there is some cross reactivity between wasp and bee venom proteins. Hyaluronidase shows the most sequence homology and MHC-II cross-reactivity can be detected, but doesn't correlate with clinical reactivity.

5. Why did the authors measure during IT the response for IFNg by ELISPOT and IL-13 and IL-10 production in supernatant? Choosing either one of the methods would allow for a IFN-g/IL-4 ratio that you expect to see changing in favor of IFNg during IT. No wit shows that T cell reactivity is enhance during the middle part of IT with an increase in both IFNg and IL-13 without any effect on IL-10. The latter is on the detection limit of the ELISA, I guess, so does not add anything.

IL-13 and L-10 levels were measured by ELISA in supernatants due to the limited number of cells available from each patient at each time point. IL-10 was measured as there has been previous interest in IL-10 playing a key role in the induction of clinical tolerance. The detection limit of the ELISA was 2-300 pg/ml, and so although the levels measured here were low, they were still within the limits of the assay and show that in this assay system, IL-10 production by CD1a-restricted T cells did not change over the course of immunotherapy.

6. In the discussion the authors explain the functions of IFNg, IL-13 and GM-CSF but how do the levels of cytokine production compare to the production of these cytokines by MHC-II restricted responses. Do these amounts contribute?

Our study was designed to rule in or out CD1a mediated responses for the first time, so most experiments were designed to isolate CD1a-mediated effects from those of MHC-reactive T cells. We share interest in the question and can say that previous studies have shown that MHC-II restricted T cells produce cytokines, including IFNγ and IL-13 in response to venom. Levels of Th2 cytokines produced are higher in allergics compared to healthy controls. We have studied the levels of MHC-II restricted cytokine producing cells in response to wasp venom from the same cohort (Aslam et al PLOS One 2010 5(6): e11028) in which we showed that the peptide-specific cells required expansion with antigen before being detectable, in contrast

to the CD1a-restricted T cells which are detectable without an antigen-specific expansion step. Indeed this is a fairly usual aspect of the CD1 response in which high precursor frequencies allow rapid response and high cytokine levels in comparison to MHC-mediated response (De Jong, Nature Immunol, 2010; Godfrey, Rossjohn, Uldrich, McCluskey, Moody; Nature Immunol, in press in 2015). Furthermore given the enrichment of CD1a expression in the skin, this suggests that CD1a-reactive T cells play a more important role in the local immune response, for example in the recruitment, activation and enhancement of the peptide specific immune response. This point has been added to the discussion.

7. In several parts of the manuscripts the authors state that CD1a is most abundantly expressed in the epidermis of the skin. However, there is also a substantial population of CD1a+ dermal DCs, and CD1a expression is also present on DC is in the respiratory tract. The numbers of CD1a+ LCs is always lower the CD1a+ DDCs and in all crawl-out skin models. This remark should be placed with more caution.

In published (Yakimchuk, EJI, 2011: De Jong, Nature Immunol 2010) and unpublished studies from the Moody lab, CD1a staining is higher in the epidermis than dermis. Also LCs (CD1a+) are generally more abundant than dermal DCs (CD1a+CD1b+CD1c+) so the statement is supported. We very much appreciate the reviewer’s comments and entirely agree that dermal DCs may also be relevant to this system where venom is delivered to the skin. We have clarified the manuscript accordingly.

8. What is the nature of the K562 cell line that is used? Can this cell line process antigens like dendritic cells or are fragments presented in the CD1a molecules? Is this presentation also happening with skin-derived CD1a+ dendritic cells? Not monocyte-derived DCs as it is now clear that many processes are not as potent with ex vivo DCs compare to the Mo-DC models.

K562 cells are of the erythroleukaemic origin used in order to study and compare cohorts of genetically unrelated individuals due to low expression of MHC by K562 cells. We entirely agree that the reviewer raises an important question and for this reason have previously shown that K562 cells accurately reflect the CD1a presentation capacity of skin derived CD1a-positive cells, as well as monocyte-derived CD1a-positive cells and CD34-derived Langerhans cell-like populations. The referee’s question is specifically addressed in Figure 1 of De Jong in Nature Immunol, 2010. In Bourgeois JExpMed 2015 we compared K562, native LCs and in vitro derived LCs. We have clarified this in the current manuscript so that readers can be aware that the question has been addressed previously. We also have added data using a CD1a-reactive T cell-line and CD1a+ cells isolated from the skin as APCs, which we have now included (Fig 4B, right panel).

Reviewer: 2:

Comments to the Author: This manuscript by Subramanian et al. shows that venom-responsive CD1a-reactive T cells are increased in the blood of venom allergic individuals. These cells are cross-reactive between bee and wasp-derived venoms, probably through the generation of similar self-lipid antigens by the phospholipase A2 activity of both venoms. Furthermore, the authors show that following subcutaneous immunotherapy with increasing amount of venom, the frequency of venom-responsive CD1a-reactive T cells diminishes. These are new and interesting data that have implications for allergy and immunotherapy approaches. The experiments are properly performed and presented.

Comments: In Fig 4, it would important to show that the cross-reactive responses can be blocked specifically by the addition of anti-CD1a mAbs.

We appreciate the reviewer's comment and have added new data in Fig 4A for cell lines generated in the presence of PLA2, showing inhibition by anti-CD1a antibody.

Reviewer: 3:

Comments to the Author: This work describes T cell responses to neolipid antigens generated by bee and wasp venom phospholipase in non-allergic and allergic individuals as well as patients treated with allergen-specific immunotherapy. The approach is technically sound and the paper is concise and well written. The conclusions are supported by the data presented.

How did the authors evaluate the induction of tolerance in their patients, coinciding with the effects on CD1a-reactive T cells? (lines 292). It should be demonstrated that the patients in this study responded to treatment by tolerance induction.

Induction of clinical tolerance was determined as the ability to tolerate the equivalent of two sting doses of wasp venom (100µg) and all patients had achieved clinical tolerance by week 8. This has been added to the manuscript.

Minor comments: The information given in the discussion (lines 243-249) on the formation of neolipids by PLA should already be given in the introduction.

Thank you, we have revised the Introduction to reflect the formation of neolipid antigens by PLA.

The IL-10 levels measured in cells from treated patients are very low, possibly below the limit of detection. What is the sensitivity limit of the applied assays?

Although the levels of IL-10 detected were low, the sensitivity of IL-10 ELISA kit used was 2-300pg/ml, suggesting that in this assay system, IL-10 produced by CD1a-reactive T cells does not change over the course of immunotherapy.

European Journal of Immunology

Second Editorial Decision - 05-Oct-2015

Dear Mrs. Subramaniam,

It is a pleasure to provisionally accept your manuscript entitled "Elevated and cross-responsive CD1a-reactive T cells in bee and wasp venom allergic individuals" for publication in the European Journal of Immunology.

For final acceptance, please follow the instructions below and return the requested items as soon as possible as we cannot process your manuscript further until all items listed below are dealt with.

Please note that EJI articles are now published online a few days after final acceptance (see Accepted Articles: http://onlinelibrary.wiley.com/journal/10.1002/(ISSN)1521-4141/accepted). The files used for the Accepted Articles are the final files and information supplied by you in Manuscript Central. You should therefore check that all the information (including author names) is correct as changes will NOT be permitted until the proofs stage.

We look forward to hearing from you and thank you for submitting your manuscript to the European Journal of Immunology.

Yours sincerely,
Laura Soto Vazquez

on behalf of
Prof. Maria Yazdanbakhsh

Editorial Office
European Journal of Immunology
e-mail: ejied@wiley.com
www.eji-journal.eu
